# The Critical Role of the Right Dorsal and Ventral Anterior Insula in Reciprocity: Evidence From the Trust and Ultimatum Games

**DOI:** 10.3389/fnhum.2020.00176

**Published:** 2020-05-15

**Authors:** Frank Krueger, Gabriele Bellucci, Pengfei Xu, Chunliang Feng

**Affiliations:** ^1^Department of Psychology, George Mason University, Fairfax, VA, United States; ^2^School of Systems Biology, George Mason University, Fairfax, VA, United States; ^3^Max Planck Institute for Biological Cybernetics, Tübingen, Germany; ^4^Shenzhen Key Laboratory of Affective and Social Neuroscience, Center for Brain Disorders and Cognitive Sciences, Shenzhen University, Shenzhen, China; ^5^Center for Neuroimaging, Shenzhen Institute of Neuroscience, Shenzhen, China; ^6^Great Bay Neuroscience and Technology Research Institute, Kwun Tong, Hong Kong; ^7^Guangdong Provincial Key Laboratory of Mental Health and Cognitive Science, Center for Studies of Psychological Application, School of Psychology, South China Normal University, Guangzhou, China

**Keywords:** trust, reciprocity, insula, decision-making, network

Social norms represent a fundamental grammar of social interactions, as they refer to shared expectations about behaviors of one's social group members (Bicchieri, 1990, 2005; Santos et al., 2018). Based on these expectations, particularly accurate predictions about another person's future behavior are possible—establishing the preconditions for cooperative interactions. Overall, group prosperity is enhanced when all members comply with social norms (i.e., *norm compliance*). However, social norms need to be enforced by sanctioning violators (i.e., *norm enforcement*). For instance, expectations of compliance with a norm of reciprocity may help overcome the fear of being betrayed by a social partner. As cooperation allows for better collective solutions than those attained by self-interested individuals, social groups are interested in enforcing compliance with social norms by their members, and developing tools for successful recognition of norm violators (Fehr and Schurtenberger, 2018). Thus, a fragile balance between incentives for norm enforcement and deterrents for sanctions of violators is required for a well-functioning society.

Interactive economic games, such as the trust game (TG) (Berg et al., 1995) and the ultimatum game (UG) (Güth et al., 1982), provide reliable experimental settings for the investigation of motivational, affective, and socio-cognitive processes involved in social norm compliance and enforcement (Corradi-Dell'acqua et al., 2016; Feng et al., 2017; Engelmann et al., 2019; Krueger and Meyer-Lindenberg, 2019). Based on the learned and internalized social norms, an agent's reciprocal behavior is determined by the evaluation of the expected or experienced kindness of a partner by weighting the partner's intentions (i.e., the underlying motivation in performing an action) and the action outcomes (i.e., positive or negative consequences of an action for oneself and others) (Falk and Fischbacher, 2006).

Recent work has shown that individuals integrate this information into their beliefs about another person's character traits for reliable predictions of the other's most likely behavior in a new social interaction (Krueger et al., 2009; Bellucci et al., 2019b; Dorfman et al., 2019). Hence, reliably estimating the kindness/unkindness of a partner facilitates norm compliance (i.e., positive reciprocity) or norm enforcement (i.e., negative reciprocity) across contexts and time. Importantly, the ability to learn from feedback about a partner's intentions and action outcomes heavily hinges on the degree to which feedback information violates one's priors and expectations (Fouragnan et al., 2013; Dorfman et al., 2019; Bellucci and Park, 2020). The ability to detect expectancy violations might even counteract biases in belief updating about another person's benevolence or malevolence.

Integrating neuroimaging data from economic games across a plethora of neuroimaging studies via coordinate-based meta-analyses (Feng et al., 2015; Bellucci et al., 2017a)—in combination with task-based and task-free functional connectivity analyses (Gurevitch et al., 2018)—has revealed the right anterior insula (R AI) as a candidate brain region for detection of norm deviations in trusting (i.e., trust game) and fairness-related (i.e., ultimatum game) interactions (Krueger et al., 2008; Bellucci et al., 2018). Representing a posterior-to-anterior remapping of interoceptive signals within the insular cortex, the R AI takes a crucial role in salience detection across multiple domains, whereas the posterior insular cortex mediates sensorimotor processes (Craig, 2009). Being part of the salience network (SAN), two functionally distinct brain regions within the R AI—a dorsal AI (dAI) and ventral AI (vAI) cluster—have been identified Kelly et al., 2012; Chang et al., 2013; Wager and Barrett, 2017). Whereas the R dAI act as a switch that exerts direct influences on the central executive network (CEN, i.e., cognitive control system, including high-order executive functions; Seeley et al., 2007; Bressler and Menon, 2010; Menon, 2011; Sheffield et al., 2015 and the default-mode network (DMN, i.e., social cognition system, including autobiographical memory, self-monitoring, and theory of mind; Andrews-Hanna et al., 2010; Bressler and Menon, 2010; Menon, 2011), the R vAI exerts direct influence on limbic cortices (which mediate affective processes) (Sridharan et al., 2008; Goulden et al., 2014; Uddin et al., 2014). These AI subregions—encoding a *common currency of aversion*—were both found consistently activated for responses to unfair behavior but differently engaged by trust and trustworthiness behaviors (Bellucci et al., 2018). In particular, the dAI was preferentially engaged by trust behavior while the vAI by trustworthiness behavior (Bellucci et al., 2018). We propose that consistent recruitment of the AI during those social behaviors is a signature of their common neural processing related to expectancy violation in the form of deviations from social norms. In particular, social behaviors in the TG and UG, such as trust in unknown partners, trustworthiness during repeated interactions and rejection of unfair offers, imply violations of two fundamental social norms —fairness and reciprocation. With this respect, they require evaluations of intentions and outcomes of actions that are aligned with individual expectations in case of compliant behaviors but that deviate from individual expectations in case of violations.

When interacting with a stranger in a one-shot TG, in which the investor interacts only once with a trustee, investors feel compelled to comply with a fairness norm and share some fair amount with the trustee. However, the probability that the trustee, whose reputation and past social behavior are supposedly unknown, betrays trust in these circumstances is not negligible. Behavioral studies have repeatedly shown that individuals in these situations worry about a hypothetical, but not much unlikely, defection to occur (Mccabe et al., 1998; Bohnet and Zeckhauser, 2004; Ashraf et al., 2006; Bohnet et al., 2008; Aimone and Houser, 2011, 2013). Individuals might hence begin prospecting to decide whether to trust, for instance, by thinking about what would be most likely that the partner thinks about compliance with a reciprocity norm, and about the reasons for which the partner would consider convenient to violate this norm—processes that likely require the recruitment of the dAI. In iterative interactions, on the contrary, individuals are likely to base their trust decisions on what they have learned from the partner over multiple encounters, switching to a more automatic, knowledge-based decision-making process involving social affiliation regions (Krueger et al., 2007). This is further consistent with the absence of AI signaling during iterative trust decisions with the same partner (Bellucci et al., 2017a).

Reciprocation of trust requires similar evaluations of norm-deviant behaviors by the trustee in a multi-round TG. The concerns that investors have from a second-person perspective, trustees have those from a first-person perspective. In particular, trustees have to weigh the advantages and disadvantages of a cooperative and non-cooperative response to the investor's kind behavior. Also, as the amount of money entrusted by investors in the TG is multiplied by a predetermined factor (usually, tripled), trustees are in an advantageous situation in which defection lures with its convenience. However, defection also implies the violation of a reciprocation norm that will enforce inequality in the payoff distribution between investors and trustees. Hence, trustees might feel guilty of taking advantage of their situation and might fear of what the partner could think of them, especially in iterative interactions where future encounters loom and the importance of a good reputation is more pressing. These aversive feelings are likely encoded in the vAI. On the contrary, in circumstances of low external incentives, such as during reciprocal decisions in single interactions where concerns about what others might think and the pressure of social norm compliance are absent, cognitive control might be required to enact reciprocity. This nicely chimes with the recruitment of dorsolateral prefrontal regions during trustworthiness behavior in single and anonymous interactions (Knoch et al., 2006; Van Den Bos et al., 2011; Nihonsugi et al., 2015).

The receiver in the UG, who faces an unfair offer from the proposer, is in a situation that likely elicits similar psychological processes to those evoked by both investors' and trustees' concerns in the TG. On the one hand, the receiver is confronted with an actual violation of the fairness norm perpetrated by the proposer who sent an unfair offer. Unfair offers elicit negative feelings (e.g., increases in skin conductance activity) in receivers who respond by rejecting the offer. Since the unfair offer implies an actual inequal outcome in resource distributions (given that unfair offers are generally lower than one-third of the resources available to proposers), the receiver might be concerned about the inequality derived from the norm violation. Outcome inequality might hence evoke negative feelings in the receiver that support negative reciprocity via recruitment of the vAI. On the other hand, however, high rejection rates and increased skin conductance activity have been observed only for unfair offers proposed by a human partner, but not for unfair offers generated by computers (Sanfey et al., 2003; Van 'T Wout et al., 2006). These results suggest that the receiver in the UG is further concerned about the intentions of the proposer and is determined to forgo immediate benefits to enforce a fairness norm via a rejection of the offer, which likely recruits the dAI.

Hence, consistent activations of the AI in all these behaviors likely refer to general signaling of violations of expectations about actions that deviate from social norms. However, given the different activation patterns of the dAI and vAI, we here propose an overarching framework in which the R AI—part of the salience network (SAN)—recruits other large-scale brain networks to determine the appropriate reciprocal behavior (via the central-executive network, CEN) based on evaluations about the partner's kindness (via the default-mode network, DMN) (Krueger and Hoffman, 2016; Bellucci et al., 2019a). Hereby, the R AI subregions play a crucial role in signaling how a deviation has occurred, in particular, because of an intentional action (R dAI) or due to an action outcome (R vAI; Figure 1).

**Figure 1:**
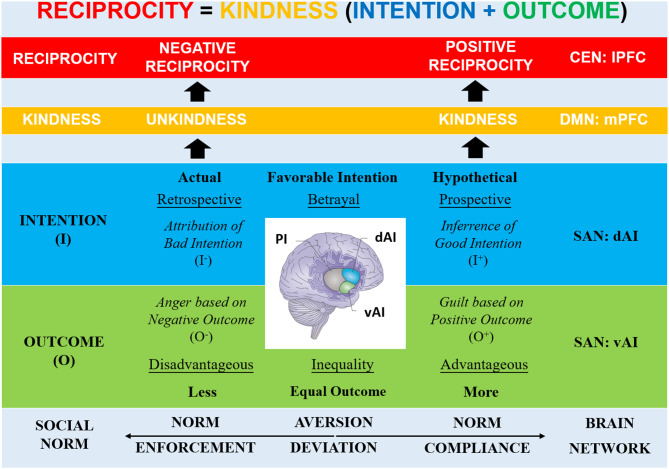
Framework: Role of R dAI and R vAI in Reciprocity. Based on social norms, an agent's reciprocal behavior is determined by evaluating the expected or experienced kindness/unkindness of a partner's normative action: the intention as the underlying motivation and the outcome as the consequence of the action. The R AI (part of SAN) recruits other large-scale networks to determine the appropriate reciprocity (e.g., lPFC via CEN) based on kindness evaluations (e.g., mPFC via DMN). The R AI subregions play a crucial role in signaling deviations from expectations on outcomes (R vAI) and intentions (R dAI) of an action, facilitating norm compliance (positive reciprocity), and norm enforcement (negative reciprocity). The vAI signals violations of expected outcomes (disadvantageous vs. advantageous outcome inequality) that elicit aversive feelings (anger vs. guilt). The dAI signals violations of expected intentional behaviors (actual vs. hypothetical betrayal) that evoke social-cognitive processes (attribution vs. inference) [Note that brain image adopted from Uddin (2015)]. R, right; SAN, Salience Network; PI, Posterior Insula; AI, Anterior Insula; vAI, Ventral Anterior Insula; dAI, Dorsal Anterior Insula; DMN, Default-mode Network; mPFC, Medial Prefrontal Cortex; CEN, Central-executive Network, lPFC, Lateral Prefrontal Cortex; O+, Positive Outcome; O-, Negative Outcome; I-, Negative Intention; I+, Positive Intention.

We propose that the SAN detects (vAI) and generates an aversive experience based on the salience of the social norm violation and provides an emotional signal (amygdala) encoding the severity of outcome related to the norm violation (Buckholtz et al., 2008). The DMN anchored in the medial prefrontal cortex (mPFC) integrates the outcome (via the ventromedial PFC's inter-network connectivity with SAN) and the intention (via the dorsomedial PFC's intra-network connectivity with the temporoparietal junction, TPJ) of the norm violation into an assessment of kindness (Krueger et al., 2009). The CEN anchored in lateral PFC (lPFC) converts the kindness signal from the DMN into an appropriate reciprocal behavior that fits the norm violation. Previous work has demonstrated that connectivity between the mPFC and lPFC was associated with evaluations of norm violations for appropriate punishment decisions (Bellucci et al., 2017b).

Therefore, in the social settings of the economic paradigms here considered, the vAI likely represents forms of *violations of an expected outcome* such as outcome inequalities (i.e., less- vs. more-than-equal) that elicit negative feelings via co-activation of the limbic network (e.g., amygdala). In particular, less-than-equal outcomes refer to a situation of *disadvantageous inequality* that triggers negative feelings such as anger and envy (due to a negative outcome for the self), which support norm enforcement in the form of negative reciprocity (e.g., punishment). On the contrary, more-than-equal outcomes refer to situations of *advantageous inequality* that likely triggers different negative feelings such as guilt (due to a positive outcome for the self), which compel to norm compliance in the form of positive reciprocity (e.g., cooperation).

The dAI, instead, likely represents forms of *violations of an expected intentional behavior* such as betrayal (both actual and hypothetical) that elicit social-cognitive processes via co-activation of the default-mode network. In particular, actual deviant behaviors prompt to retrospection on the intentionally perpetrated betrayal that triggers socio-cognitive processes such as attribution of bad intentions, thereby promoting norm enforcement in the form of negative reciprocity (e.g., punishment). On the contrary, hypothetical deviant behaviors prompt to prospection on a possible intentional betrayal that triggers socio-cognitive processes such as inferences on the other's intentions, thereby supporting norm compliance in the form of positive reciprocity (e.g., trust).

Given the proposed neuropsychological model, some predictions for other recently reported activation patterns associated with social normative behaviors are possible. For instance, social interactions in which some form of expectancy violation is involved might require recruitment of the AI. For the classical Prisoner's Dilemma game, where two players can decide to cooperate or betray each other, both parties—acting in their own self-interests—choose often to protect themselves at the expense of the other player; thereby, producing the worst outcome for both parties by non-reciprocation of cooperation (Peterson, 2015). A neuroimaging study employing an iterated version of the Prisoner's Dilemma game showed greater activation in R dAI during unreciprocated compared to reciprocated cooperation when both players were informed about the outcome of each trial game (but not during their decisions) (Rilling et al., 2008). Another study revealed that depressed compared to healthy individuals reported higher levels of negative feelings (i.e., betrayal, guilt) during this game. Across all players, the R vAI was more activated comparing outcomes, where one of the players cooperated and the other defected, with outcomes, where both players either cooperated or defected (Gradin et al., 2016).

Further, shame and embarrassment, which emerge from the recognition that one's behavior diverges from a group's expectancies, should elicit activations in the AI. Preliminary evidence aligns with this prediction and suggests that shame and embarrassment elicit activations particularly in the vAI, consistently with the fact that these negative feelings are based on violations caused by the consequences (and not the intentions) of one's behavior (Muller-Pinzler et al., 2015; Zhu et al., 2019). Similarly, punishment and blame, which rely on the recognition of another's deviant behavior, should recruit the AI as well. Previous evidence chimes with this prediction, pointing specifically to the dAI, consistently with the fact that punishment and blame require socio-cognitive processes for understanding reasons and motives of another's wrongdoing (Krueger and Hoffman, 2016; Patil et al., 2017; Bellucci et al., 2020). On the contrary, other social behaviors such as generosity or altruism should activate the AI only if they also involve expectancy violations. Previous work on these behaviors seems to confirm such prediction (Moll et al., 2006; Coll et al., 2017; Karns et al., 2017), showing AI activations only when a form of expectancy violation is involved such as when helping an offender or breaking a promise to cooperate (Baumgartner et al., 2009; David et al., 2017).

Taken together, the AI is an underestimated but essential brain region for understanding human social cognition and its pathophysiological forms in social brain disorders such as schizophrenia and autism (Namkung et al., 2017). Our framework provides a distinctive mapping of the R AI subdivisions that can be employed in future multimodal neuroimaging studies to test hypotheses on the AI functioning in reciprocity. For this reason, our neuropsychological framework contributes to a more comprehensive understanding of this region for basic and clinical neuroscience in which altered processing in AI subdivisions determine different aspects of prevalent brain disorders (e.g., psychosis, autism).

## Author Contributions

FK and CF prepared the first draft of the article. All authors contributed to the final version.

## Conflict of Interest

The authors declare that the research was conducted in the absence of any commercial or financial relationships that could be construed as a potential conflict of interest.

## References

[B1] Aimone J. A. Houser D. (2011). Beneficial betrayal aversion. PLoS ONE 6:e17725. 10.1371/journal.pone.0017725 21423732 PMC3056706

[B2] Aimone J. A. Houser D. (2013). Harnessing the benefits of betrayal aversion. J. Economic Behav. Organiz. 89, 1–8. 10.1016/j.jebo.2013.02.001

[B3] Andrews-Hanna J. R. Reidler J. S. Sepulcre J. Poulin R. Buckner R. L. (2010). Functional-anatomic fractionation of the brain's default network. Neuron 65, 550–562. 10.1016/j.neuron.2010.02.005 20188659 PMC2848443

[B4] Ashraf N. Bohnet I. Piankov N. (2006). Decomposing trust and trustworthiness. Exp. Econom. 9, 193–208. 10.1007/s10683-006-9122-4

[B5] Baumgartner T. Fischbacher U. Feierabend A. Lutz K. Fehr E. (2009). The neural circuitry of a broken promise. Neuron 64, 756–770. 10.1016/j.neuron.2009.11.017 20005830

[B6] Bellucci G. Camilleri J. A. Iyengar V. Eickhoff S. B. Krueger F. (2020). The emerging neuroscience of social punishment: meta-analytic evidence. Neurosci. Biobehav. Rev. 6:e1000097 10.1016/j.neubiorev.2020.04.011 PMC7291369 32302599

[B7] Bellucci G. Chernyak S. Hoffman M. Deshpande G. Dal Monte O. Knutson K. M.. (2017b). Effective connectivity of brain regions underlying third-party punishment: functional MRI and Granger causality evidence. Soc. Neurosci. 12, 124–134. 10.1080/17470919.2016.1153518 26942651

[B8] Bellucci G. Chernyak S. V. Goodyear K. Eickhoff S. B. Krueger F. (2017a). Neural signatures of trust in reciprocity: a coordinate-based meta-analysis. Hum Brain Mapp 38, 1233–1248. 10.1002/hbm.23451 27859899 PMC5441232

[B9] Bellucci G. Feng C. Camilleri J. Eickhoff S. B. Krueger F. (2018). The role of the anterior insula in social norm compliance and enforcement: evidence from coordinate-based and functional connectivity meta-analyses. Neurosci. Biobehav. Rev. 92, 378–389. 10.1016/j.neubiorev.2018.06.024 29958872

[B10] Bellucci G. Hahn T. Deshpande G. Krueger F. (2019a). Functional connectivity of specific resting-state networks predicts trust and reciprocity in the trust game. Cogn. Affect. Behav. Neurosci. 19, 165–176. 10.3758/s13415-018-00654-3 30357662

[B11] Bellucci G. Molter F. Park S. Q. (2019b). Neural representations of honesty predict future trust behavior. Nat. Commun. 10:5184. 10.1038/s41467-019-13261-8 31729396 PMC6858375

[B12] Bellucci G. Park S. Q. (2020). Honesty biases trustworthiness impressions. J. Exp. Psychol. Gen. 10.1037/xge0000730. [Epub ahead of print]. 31916837

[B13] Berg J. Dickhaut J. Mccabe K. (1995). Trust, reciprocity, and social history. Games Economic Behav. 10, 122–142. 10.1006/game.1995.1027

[B14] Bicchieri C. (1990). Norms of cooperation. Ethics 100, 838–861. 10.1086/293237

[B15] Bicchieri C. (2005). The Grammar of Society: The Nature and Dynamics of Social Norms. Cambridge: Cambridge University Press.

[B16] Bohnet I. Greig F. Herrmann B. Zeckhauser R. (2008). Betrayal aversion: evidence from Brazil, China, Oman, Switzerland, Turkey, and the United States. Am. Economic Rev. 98, 294–310. 10.1257/aer.98.1.294

[B17] Bohnet I. Zeckhauser R. (2004). Trust, risk and betrayal. J. Economic Behav. Organization 55, 467–484. 10.1016/j.jebo.2003.11.004

[B18] Bressler S. L. Menon V. (2010). Large-scale brain networks in cognition: emerging methods and principles. Trends Cogn. Sci. 14, 277–290. 10.1016/j.tics.2010.04.004 20493761

[B19] Buckholtz J. W. Asplund C. L. Dux P. E. Zald D. H. Gore J. C. Jones O. D.. (2008). The neural correlates of third-party punishment. Neuron 60, 930–940. 10.1016/j.neuron.2008.10.016 19081385

[B20] Chang L. J. Yarkoni T. Khaw M. W. Sanfey A. G. (2013). Decoding the role of the insula in human cognition: functional parcellation and large-scale reverse inference. Cereb. Cortex 23, 739–749. 10.1093/cercor/bhs065 22437053 PMC3563343

[B21] Coll M. P. Gregoire M. Eugene F. Jackson P. L. (2017). Neural correlates of prosocial behavior towards persons in pain in healthcare providers. Biol. Psychol. 128, 1–10. 10.1016/j.biopsycho.2017.06.005 28669784

[B22] Corradi-Dell'acqua C. Tusche A. Vuilleumier P. Singer T. (2016). Cross-modal representations of first-hand and vicarious pain, disgust and fairness in insular and cingulate cortex. Nat. Commun. 7:10904. 10.1038/ncomms10904 26988654 PMC4802033

[B23] Craig A. D. (2009). How do you feel–now? The anterior insula and human awareness. Nat. Rev. Neurosci. 10, 59–70. 10.1038/nrn2555 19096369

[B24] David B. Hu Y. Kruger F. Weber B. (2017). Other-regarding attention focus modulates third-party altruistic choice: An fMRI study. Sci. Rep. 7:43024. 10.1038/srep43024 28220867 PMC5318960

[B25] Dorfman H. M. Bhui R. Hughes B. L. Gershman S. J. (2019). Causal inference about good and bad outcomes. Psychol. Sci. 30, 516–525. 10.1177/0956797619828724 30759048 PMC6472176

[B26] Engelmann J. B. Meyer F. Ruff C. C. Fehr E. (2019). The neural circuitry of affect-induced distortions of trust. Sci. Adv. 5:eaau3413. 10.1126/sciadv.aau3413 30891491 PMC6415955

[B27] Falk A. Fischbacher U. (2006). A theory of reciprocity. Games Economic Behav. 54, 293–315. 10.1016/j.geb.2005.03.001

[B28] Fehr E. Schurtenberger I. (2018). Normative foundations of human cooperation. Nat. Hum. Behav. 2, 458–468. 10.1038/s41562-018-0385-5 31097815

[B29] Feng C. Azarian B. Ma Y. Feng X. Wang L. Luo Y. J. (2017). Mortality salience reduces the discrimination between in-group and out-group interactions: a functional MRI investigation using multi-voxel pattern analysis. Hum. Brain Mapp. 38, 1281–1298. 10.1002/hbm.23454 27859936 PMC6866768

[B30] Feng C. Luo Y.-J. Krueger F. (2015). Neural signatures of fairness-related normative decision making in the ultimatum game: a coordinate-based meta-analysis. Hum. Brain Mapping 36, 591–602. 10.1002/hbm.22649 25327760 PMC6869807

[B31] Fouragnan E. Chierchia G. Greiner S. Neveu R. Avesani P. Coricelli G. (2013). Reputational priors magnify striatal responses to violations of trust. J. Neurosci. 33, 3602–3611. 10.1523/JNEUROSCI.3086-12.2013 23426687 PMC6619519

[B32] Goulden N. Khusnulina A. Davis N. J. Bracewell R. M. Bokde A. L. Mcnulty J. P.. (2014). The salience network is responsible for switching between the default mode network and the central executive network: replication from DCM. Neuroimage 99, 180–190. 10.1016/j.neuroimage.2014.05.052 24862074

[B33] Gradin V. B. Perez A. Macfarlane J. A. Cavin I. Waiter G. Tone E. B.. (2016). Neural correlates of social exchanges during the Prisoner's Dilemma game in depression. Psychol. Med. 46, 1289–1300. 10.1017/S0033291715002834 26763141

[B34] Gurevitch J. Koricheva J. Nakagawa S. Stewart G. (2018). Meta-analysis and the science of research synthesis. Nature 555, 175–182. 10.1038/nature25753 29517004

[B35] Güth W. Schmittberger R. Schwarze B. (1982). An experimental analysis of ultimatum bargaining. J. Economic Behav. Organiz. 3, 367–388. 10.1016/0167-2681(82)90011-7

[B36] Karns C. M. Moore W. E. III. Mayr U. (2017). The cultivation of pure altruism via gratitude: a functional MRI study of change with gratitude practice. Front. Hum. Neurosci. 11:599. 10.3389/fnhum.2017.00599 29375336 PMC5770643

[B37] Kelly C. Toro R. Di Martino A. Cox C. L. Bellec P. Castellanos F. X.. (2012). A convergent functional architecture of the insula emerges across imaging modalities. Neuroimage 61, 1129–1142. 10.1016/j.neuroimage.2012.03.021 22440648 PMC3376229

[B38] Knoch D. Pascual-Leone A. Meyer K. Treyer V. Fehr E. (2006). Diminishing reciprocal fairness by disrupting the right prefrontal cortex. Science 314, 829–832. 10.1126/science.1129156 17023614

[B39] Krueger F. Barbey A. K. Grafman J. (2009). The medial prefrontal cortex mediates social event knowledge. Trends Cognitive Sci. 13, 103–109. 10.1016/j.tics.2008.12.005 19223228

[B40] Krueger F. Grafman J. Mccabe K. (2008). Neural correlates of economic game playing. Philos. Trans. R Soc. Lond. B Biol. Sci. 363, 3859–3874. 10.1098/rstb.2008.0165 18829425 PMC2581786

[B41] Krueger F. Hoffman M. (2016). The emerging neuroscience of third-party punishment. Trends Neurosci. 39, 499–501. 10.1016/j.tins.2016.06.004 27369844

[B42] Krueger F. Mccabe K. Moll J. Kriegeskorte N. Zahn R. Strenziok M.. (2007). Neural correlates of trust. Proc. Natl. Acad. Sci. U.S.A. 104, 20084–20089. 10.1073/pnas.0710103104 18056800 PMC2148426

[B43] Krueger F. Meyer-Lindenberg A. (2019). Toward a model of interpersonal trust drawn from neuroscience, psychology, and economics. Trends Neurosci. 42, 92–101. 10.1016/j.tins.2018.10.004 30482606

[B44] Mccabe K. A. Rassenti S. J. Smith V. L. (1998). Reciprocity, trust, and payoff privacy in extensive form bargaining. Games Econ. Behav. 24, 10–24. 10.1006/game.1998.0638

[B45] Menon V. (2011). Large-scale brain networks and psychopathology: a unifying triple network model. Trends Cogn. Sci. 15, 483–506. 10.1016/j.tics.2011.08.003 21908230

[B46] Moll J. Krueger F. Zahn R. Pardini M. De Oliveira-Souza R. Grafman J. (2006). Human fronto-mesolimbic networks guide decisions about charitable donation. Proc. Natl. Acad. Sci. U.S.A. 103, 15623–15628. 10.1073/pnas.0604475103 17030808 PMC1622872

[B47] Muller-Pinzler L. Gazzola V. Keysers C. Sommer J. Jansen A. Frassle S.. (2015). Neural pathways of embarrassment and their modulation by social anxiety. Neuroimage 119, 252–261. 10.1016/j.neuroimage.2015.06.036 26093329 PMC5008438

[B48] Namkung H. Kim S. H. Sawa A. (2017). The insula: an underestimated brain area in clinical neuroscience, psychiatry, and neurology. Trends Neurosci. 40, 200–207. 10.1016/j.tins.2017.02.002 28314446 PMC5538352

[B49] Nihonsugi T. Ihara A. Haruno M. (2015). Selective increase of intention-based economic decisions by noninvasive brain stimulation to the dorsolateral prefrontal cortex. J. Neurosci. 35, 3412–3419. 10.1523/JNEUROSCI.3885-14.2015 25716841 PMC6605550

[B50] Patil I. Calo M. Fornasier F. Cushman F. Silani G. (2017). The behavioral and neural basis of empathic blame. Sci. Rep. 7:5200. 10.1038/s41598-017-05299-9 28701703 PMC5508012

[B51] Peterson M. (2015). The Prisoner's Dilemma. Cambridge: Cambridge University Press.

[B52] Rilling J. K. Goldsmith D. R. Glenn A. L. Jairam M. R. Elfenbein H. A. Dagenais J. E.. (2008). The neural correlates of the affective response to unreciprocated cooperation. Neuropsychologia 46, 1256–1266. 10.1016/j.neuropsychologia.2007.11.033 18206189

[B53] Sanfey A. G. Rilling J. K. Aronson J. A. Nystrom L. E. Cohen J. D. (2003). The neural basis of economic decision-making in the Ultimatum Game. Science 300, 1755–1758. 10.1126/science.1082976 12805551

[B54] Santos F. P. Santos F. C. Pacheco J. M. (2018). Social norm complexity and past reputations in the evolution of cooperation. Nature 555, 242–245. 10.1038/nature25763 29516999

[B55] Seeley W. W. Menon V. Schatzberg A. F. Keller J. Glover G. H. Kenna H.. (2007). Dissociable intrinsic connectivity networks for salience processing and executive control. J. Neurosci. 27, 2349–2356. 10.1523/JNEUROSCI.5587-06.2007 17329432 PMC2680293

[B56] Sheffield J. M. Repovs G. Harms M. P. Carter C. S. Gold J. M. Macdonald A. W. III. Daniel Ragland J.. (2015). Fronto-parietal and cingulo-opercular network integrity and cognition in health and schizophrenia. Neuropsychologia 73, 82–93. 10.1016/j.neuropsychologia.2015.05.006 25979608 PMC4505838

[B57] Sridharan D. Levitin D. J. Menon V. (2008). A critical role for the right fronto-insular cortex in switching between central-executive and default-mode networks. Proc. Natl. Acad. Sci. U.S.A. 105, 12569–12574. 10.1073/pnas.0800005105 18723676 PMC2527952

[B58] Uddin L. Q. (2015). Salience processing and insular cortical function and dysfunction. Nat. Rev. Neurosci. 16, 55–61. 10.1038/nrn3857 25406711

[B59] Uddin L. Q. Kinnison J. Pessoa L. Anderson M. L. (2014). Beyond the tripartite cognition-emotion-interoception model of the human insular cortex. J. Cogn. Neurosci. 26, 16–27. 10.1162/jocn_a_00462 23937691 PMC4074004

[B60] Van Den Bos W. Van Dijk E. Westenberg M. Rombouts S. A. Crone E. A. (2011). Changing brains, changing perspectives: the neurocognitive development of reciprocity. Psychol. Sci. 22, 60–70. 10.1177/0956797610391102 21164174

[B61] Van 'T Wout M. Kahn R. S. Sanfey A. G. Aleman A. (2006). Affective state and decision-making in the Ultimatum Game. Exp. Brain Res. 169, 564–568. 10.1007/s00221-006-0346-5 16489438

[B62] Wager T. D. Barrett L. F. (2017). From affect to control: functional specialization of the insula in motivation and regulation. 10.1101/102368

[B63] Zhu R. Feng C. Zhang S. Mai X. Liu C. (2019). Differentiating guilt and shame in an interpersonal context with univariate activation and multivariate pattern analyses. Neuroimage 186, 476–486. 10.1016/j.neuroimage.2018.11.012 30439509

